# Thermal Warning and Shut‐down of Lithium Metal Batteries Based on Thermoresponsive Electrolytes

**DOI:** 10.1002/advs.202400953

**Published:** 2024-06-17

**Authors:** Yueyang Lan, Liujie Xiang, Junyu Zhou, Sheng Jiang, Yifan Ge, Caihong Wang, Shuai Tan, Yong Wu

**Affiliations:** ^1^ School of Chemical Engineering Sichuan University No.24 South Section 1 Yihuan Road Chengdu 610065 China

**Keywords:** batteries, LCST, overheating protection, thermal runaway, thermoresponsive electrolytes

## Abstract

The thermal runaway issue represents a long‐standing obstacle that retards large‐scale applications of lithium metal batteries. Various approaches to inhibit thermal runaway suffer from some intrinsic drawbacks, either being irreversible or delayed thermal protection. Herein, this work has explored thermo‐responsive lower critical solution temperature (LCST) ionic liquid‐based electrolytes, which provides reversible overheating protection for batteries with warning and shut‐down stages, well corresponding to an initial stage of thermal runaway process. The batteries could function stably below 70 °C as a working mode, while demonstrating a warning mode above 80 °C with a noticeable reduction in specific capacitance to delay temperature increase of batteries. In terms of 110 °C as a critically dangerous temperature, a shut‐down mode is designed to minimize the thermal energy releasing as the batteries are barely chargeable and dischargeable. Dynamically growing polymeric particles above LCST contributed to such an intelligent and mild control on specific capacitance. Larger size will occupy larger surfaces of electrodes and close more pores of separators, enabling a gradual suppressing of Li^+^ transfer and reactions. The present work demonstrated a scientific design of thermoresponsive LCST electrolytes with a superiorly precise and intelligent control of electrochemical performances to achieve self‐adapted overheating protections.

## Graphical abstract

Thermoresponsive electrolytes provide an intelligent thermal protection for lithiummetal batteries with warning and shut‐down stages, exactly corresponding to thermal runaway.

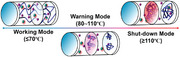

## 1 Introduction

The rapid growth of electrically powered devices requires rechargeable batteries with higher energy density and safety.^[^ ^1^, ^2^, ^3^, ^4^, ^5^ ^]^ Lithium metal batteries (LMBs) have been considered as one of the promising next‐generation rechargeable batteries due to high theoretical specific capacity (3860 mAh g^−1^) and the lowest negative redox potential (–3.04 V vs standard hydrogen electrode) of lithium metal anode. Exploring the LMBs with excellent cycling stability and high energy density has made significant progress through the scientific design of various electrodes and electrolytes.^[^ ^6^, ^7^, ^8^, ^9^ ^]^ However, the safety hazards originated from volatile, reactive, and flammable electrolytes posed a major concern for energy devices to prevent their use in large‐format systems.^[^ ^10^, ^11^, ^12^, ^13^ ^]^ The thermal abuse from over‐charging, abrupt changes in the environment, etc. would lead to thermal failure of LMBs, which could be manifested as fires or even explosions. To develop electrolytes which is non‐flammable, non‐volatile, and thermally safe at high temperatures, is important and meaningful for fabricating high‐safety LMBs.

There are three characteristic temperatures, to characterize the temperature rise in cells during thermal runaway: Li anode itself contributes to the self‐heating feature of the cells (*T*_1_), the reactions between Li metal anode and the electrolyte contribute to the occurrence of thermal runaway (*T*_2_), and exothermic reactions including electrodes and electrolyte lead to maximum temperature (*T*_3_).^[^ ^14^, ^15^ ^]^ Once thermal runaway enters the *T*_2_ stage, the LMBs reach catastrophic temperatures in a matter of seconds, which pose a formidable obstacle to any safety design to take effect. It is noted that the decomposition of solid electrolyte interphase (SEI) always occurs earlier before reaching *T*_1_ in thermal runaway.^[^ ^14^ ^]^ Therefore, to stop temperature increase at the *T*_1_ stage or even earlier temperature is imperative to construct intrinsically safe LMBs, which is highly meaningful to ensure the thermal reversibility of electrochemical performances of the LMBs.

Thermoresponsive electrolytes and electrodes have been proposed and designed to demonstrate overheating self‐protection against thermal runaway.^[^ ^16^, ^17^, ^18^ ^]^ Particularly, thermoresponsive lower critical solution temperature (LCST) electrolytes have attracted much attention for their reversible overheating protection.^[^ ^19^, ^20^, ^21^ ^]^ Poly(benzyl methacrylate)(PBnMA) demonstrated LCST in ionic liquids,^[^ ^22^ ^]^ and the PBnMA‐based ionic liquid electrolytes successfully switched overheating protection precisely above 110 °C.^[^ ^23^ ^]^ At 110 °C, the PBnMA polymer well senses the thermoresponsive temperature, and the polymer chains automatically form polymeric particles in electrolytes. Then, the particles will deposit on the surface of electrodes to reduce electrochemical reaction surfaces above 110 °C. And those polymeric nanoparticles are less conductive, they would abruptly deteriorate ion transfer process to shut down electrochemical reaction, giving an almost zero specific capacitance above 110 °C. Thus, thermoresponsive electrolytes could delay or prevent thermal runaway. However, such thermoresponsive temperatures are not in the initial stage to reach the *T*_1_. The decomposition of SEI above 110 °C negatively affects reversibility. It would be better to modulate electrochemical performances at the beginning of SEI decomposition, which will afford more time to control thermal runaway considering slow increase of temperatures at the initial stages of thermal runaway.^[^ ^14^ ^]^ Otherwise, highly accelerated reaction rates at high temperatures poses a difficulty in reducing thermal release. More importantly, an abrupt drop to zero specific capacitance above 110 °C will bring a sudden interruption of electrically powered devices, which poses a threat to the running systems. A warning stage before occurring a complete shutdown of electrochemical performances in LMBs is urgently essential. Thus, to scientifically design thermoresponsive electrolytes enabling a gradual decline of electrochemical performances of LMBs and well‐fitting to critical temperatures toward thermal runaway is highly required, yet challenging.

In view of polymer science, LCST behaviors are generally the first‐order phase separation. Above LCST, increasing temperature would dynamically rebalance particle distributions with changes in size and numbers. Higher temperature, less compatibility. The increased incompatibility between polymer and the ionic liquid electrolytes would lower the barrier to nucleation of the aggregates and enhance the rate of aggregates formation.^[^ ^24^ ^]^ Such dynamic particle distributions would be promising to affect the ion transfer and electrochemical reactions in varying degrees. It is envisioned that large size of aggregates might be able to block larger numbers of the pores in the separator and occupy much larger area on electrodes, with a result of less energy storage. Thus, a mild control of electrochemical properties in LMBs would be hopefully afforded by choosing proper thermoresponsive LCST electrolytes.

**Figure 1:**
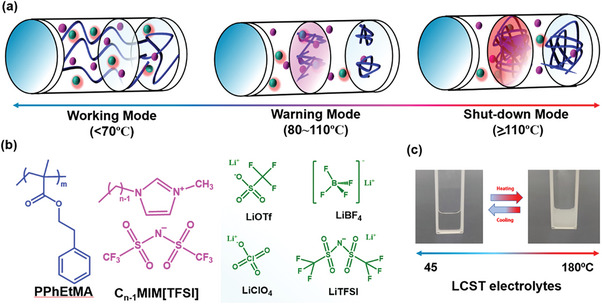
An illustration of proposed three modes for smart LMBs: working mode, warning mode, and shut‐down mode a); Chemical structures of LCST electrolytes containing PPhEtMA, C_n‐1_mim[TFSI] and Lithium salts b) and pictures of reversible LCST electrolytes with tailorable responsive temperatures from 45 to 180 °C c).

Hence, poly(phenol methacrylate) (PPhEtMA) had been chosen to investigate its thermoresponsive LCST behaviors in ionic liquid electrolytes and thermoresponsive electrochemical properties in LMBs. We previously reported that PPhEtMA systems tend to form more dynamic and soft particles, rather than frozen particles as observed in PBnMA system.^[^ ^25^ ^]^ A series of lithium salts with various anions were explored to investigate ion effect on thermoresponsive temperatures. Optimized LCST electrolytes successfully demonstrated three stages to control charge/discharge process. Based on thermoresponsive control of specific capacitance, three stages for smart LMBs were proposed: working mode (>120 mAh g, ≤70 °C), warning mode (15–120 mAh g, 80–110 °C), and shut‐down mode (<15 mAh g, ≥110 °C) as illustrated in **Figure** **1**a. The responsive temperature ranges of warning and shut down modes were well corresponding to critical process of SEI decomposition and *T*_1_ stage, respectively. A complete recovering electrochemical performances required 12 h with a resuming 97% specific capacity after a long time overheating at 110 °C. The temperature‐dependent size changes of polymeric aggregates would affect lithium transfer and insertion reactions in varing degrees. Larger aggregations would heavily block the pores of separator as well as cover active surfaces of electrodes to deteriorate performances of LMBs. The present work demonstrates that an interdisciplinary integration of stimulus polymer science into energy storage devices is highly amazing to produce a unique capability of self‐adopted overheating protection for LMBs.

## 2 Results and Discussion

Choosing the proper LCST polymer is primary to control thermoresponsive temperatures. PBnMA as a typical LCST polymers were well explored in ionic liquid systems. But a long time overheating of PBnMA aggregates at high temperatures risks an irreversible LCST behavior of PBnMA.^[^ ^25^, ^26^ ^]^ The π–π stacking in the PBnMA aggregates was too strong to allow reversible dissociation process. And thermoresponsive temperatures were generally above 110 °C, because lithium salt addition would make responsive temperature much higher, far away from the SEI decomposition temperatures.^[^ ^27^ ^]^ Hence, in terms of reversibility and critical temperatures of thermal runaway, proper LCST electrolytes need to be further explored with a well consideration of thermal runaway process of LMBs. PPhEtMA with an elongation of the alkyl chain of aromatic and ester could suppress the polymeric π–π stacking to afford rapid reversibility while providing a much lower responsive temperature at 42 °C.^[^ ^28^ ^]^ Thus, PPhEtMA was chosen as the LCST polymer to explore thermoresponsive PPhEtMA electrolytes. The PPhEtMA was polymerized via a free radial polymerization. The chemical structures of LCST electrolytes were shown in Figure 1b. Conventional salts, including LiTFSI, LiOTf, LiBF_4_, and LiClO_4_, were added to fabricate the electrolytes and the thermoresponsive temperatures were tailorable in a range of 45–180 °C as shown in Figure 1c.

**Figure 2:**
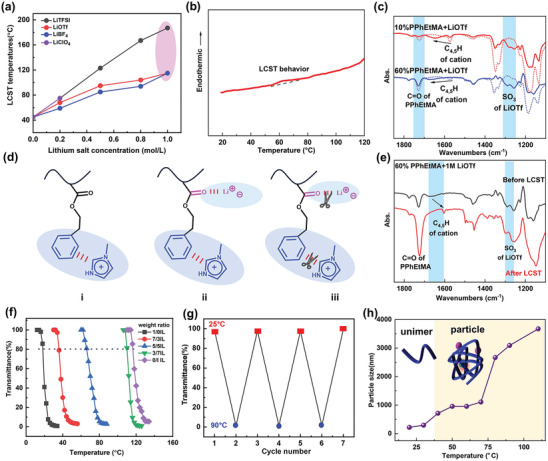
Thermoresponsive LCST temperatures of 5 wt.% PPhEtMA in C_2_MIM[TFSI] containing various contents (0.2, 0.5, 0.8, and 1.0 mol L^−1^) of lithium salts: LiTFSI, LiOTf, LiBF_4_, and LiClO_4_ a); DSC curve of 5 wt.% PPhEtMA in C_2_MIM[TFSI] containing 0.2 mol L^−1^ LiOTf upon the second heating process b); FT‐IR spectra of LCST electrolytes containing 10 wt.% and 60 wt.% PPhEtMA in presence (solid line) and absence (dot line) of LiOTf in C_2_MIM[TFSI], respectively c); An illustration of molecular interplay for 5 wt.% PPhEtMA in C_2_MIM[TFSI] in absence of LiOTf before LCST (i) and in presence of LiOTf before LCST(ii), and above LCST in presence of LiOTf (iii) d); FT‐IR spectra for 60 wt.% PPhEtMA electrolytes containing 1.0 mol L^−1^ LiOTf before and after LCST e); Transmittance measurements of 5 wt.% PPhEtMA in mixtures of C_2_MIM[TFSI] and C_1_MIM[TFSI] containing 1 mol L^−1^ LiOTf and weight ratio of C_1_MIM[TFSI]/C_2_MIM[TFSI] are 1/0, 7/3, 5/5, 3/7 and 0/1, respectively f); Repeatable cycles of LCST electrolytes switched at 25 °C and 90 °C g); Size measurements of 2PPhETMA‐5/5IL electrolytes upon heating process h).

Thermoresponsive temperatures were measured by transmittance measurements as depicted in **Figure** **2**a. As LiTFSI concentrations increased from 0 to 1.0 mol L^−1^, the values of LCST temperatures elevated from 45 to 180 °C (Figure S1, Supporting Information), while LiBF_4_ and LiOTf addition promoted LCST temperatures from 45 to 100 °C (Figures S2 andS3, Supporting Information). LiClO_4_ was not so soluble in the C_2_MIM[TFSI] and the LCST temperature of the solution containing 0.2 mol L^−1^ LiClO_4_ was as high as 78 °C. It seemed that thermoresponsive temperature were highly sensitive to lithium salts. DSC curve of 5 wt.% PPhEtMA in C_2_MIM[TFSI] containing 0.2 mol L^−1^ LiOTf demonstrated a LCST phase transition peak ≈70 °C (Figure 2b). The enthalpy values obtained from DSC curves for the LCST phase transitions were listed in Table S1 (Supporting Information) to resemble the interplay between the polymer and the ionic liquids electrolytes. The enthalpy values for LCST electrolytes containing 0.2 mol L^−1^ LiOTf, LiBF_4_, LiClO_4_, and LiTFSI were 2.26, 2.14, 2.47, and 2.50 J g^−1^, respectively. Higher enthalpy values, higher thermoresponsive temperatures. Those small enthalpy values indicated the present system was highly sensitive to the specific ion environments. Larger size of TFSI¯ might contribute to a weak bonding with Li^+^, where Li^+^ could be highly free to interplay with the polymer, as evidenced by high enthalpy values. FT‐IR measurements were further conducted to clarify the Li^+^ effect on LCST behaviors. As shown in Figure 2c, an absolute blue shift of C_4,5_H from imidazolium cation was observed from 1571 to 1649 cm^−1^, which disappeared in the range of 3207–3083 cm^−1^(Figure S4, Supporting Information) after adding LiOTf into the 5/5IL containing 10 wt.% PPhEtMA. Similar changes were also observed at high concentrations of PPhEtMA (Figure 2c; Figure S5, Supporting Information). These noticeable changes proved the presence of π‐cation interplay between PPhEtMA with imidazolium cations, which was enhanced by lithium salt addition as illustrated in Figure 2d.^[^ ^28^ ^]^ Meanwhile, increasing PPhEtMA contents induced peak intensity changes of C═O from PPhEtMA and SO_3_ from LiOTf at 1280 cm^−1^, which suggested a possible interplay between C═O and Li^+^ (Figure 2d).^[^ ^29^ ^]^ To break those interaction, much more thermal energy was required and thus an increase of thermoresponsive temperatures was observed by adding lithium salts. Upon heating process, C_4,5_H reversed to the pristine positions at 1571 cm^−1^ as well as the C═O intensity of PPhEtMA (Figure 2e). The π‐cation interplay between PPhEtMA with imidazolium cations disappeared upon heating process (Figure 2d), which dominated the occurrence of LCST behavior. To conclude, lithium salts promoted compatibility between the polymer and the ionic liquid electrolytes, contributing to an absolute increase in thermoresponsive temperatures.

However, in view of electrolytes, these thermoresponsive temperatures were too high. When the contents of lithium salts reached 1.0 mol L^−1^, LCST temperatures for PPhEtMA solutions were 180 °C (LiTFSI), 100 °C (LiOTf) and 100 °C (LiBF_4_), respectively. They were above the initial decomposition temperatures of SEI membrane. Lowering the LCST temperatures was essential and predominate for an application in thermoresponsive electrolytes. C_1_mim[TFSI] was employed to further regulate LCST temperatures. Shortening the alkyl chain length of imidazolium cation could reduce thermoresponsive temperatures in a wide range.^[^ ^30^ ^]^ 5LCST‐5/5IL means 5 wt.% PPhEtMA polymer in the ionic liquid electrolytes and 5/5IL resembles that a weight ratio of C_1_mim[TFSI]: C_2_mim[TFSI] is 5:5. As shown in Figure 2f, by increasing weight ratio of C_1_mim[TFSI]: C_2_mim[TFSI] from 1:0 to 0:1, the thermoresponsive temperatures of PPhEtMA electrolytes containing 1 mol L^−1^ LiOTf ranged from 35 to 125 °C. The thermoresponsive temperatures were highly tailorable by facilely tuning ionic liquid ratios. Particularly, with a mol ratio of C_1_mim[TFSI]: C_2_mim[TFSI] from 5:5 to 7:3, the thermoresponsive temperatures could be efficiently regulated ≈60–75 °C, which were suitable for specific and designable requirements for overheating protection.^[^ ^31^ ^]^ Switching LCST solutions at 25 and 90°C demonstrated stable thermo‐reversibility (Figure 2g). Above LCST, PPhEtMA particle size were measured as shown in Figure 2h. The particle size below 20 nm are corresponding to unimer, as an indication of the dissolution state of polymer chains in ionic liquid electrolytes. An increase of size above 40 °C resembled an occurrence of LCST to form polymeric particles. Further increasing temperature above 80 °C will greatly decrease viscosities to accelerate polymeric movements and enhance entanglement with a result of forming large particles. Figure S6 (Supporting Information) showed particle distribution at 90 and 110 °C under polarized optical microscopic observations and these larger aggregates could dissociate again when the temperature was switched to 25 °C (Figure S7, Supporting Information). Apparently, the particle size was increased upon heating process above LCST. It was expected that those particles would block the pores of separators in LMBs to influence the ion transfer process. In short, the present PPhEtMA electrolytes demonstrated tailorable thermoresponsive temperatures and thermo‐reversibility, which were particularly useful and attractive for further application in smart battery with thermoresponsive electrochemical performances.

**Figure 3:**
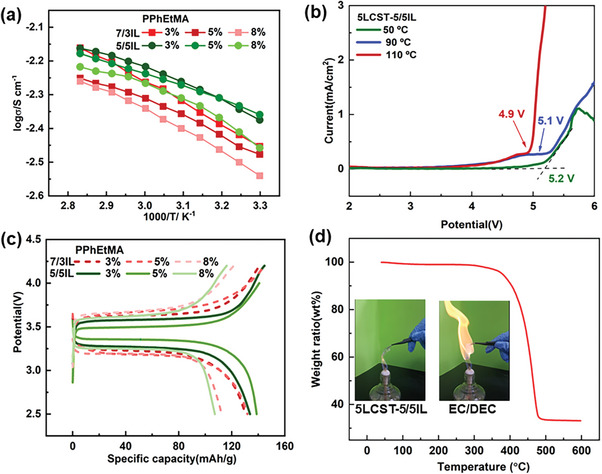
Ionic conductivities for a series of LCST electrolytes containing 3%, 5% and 8% (weight ratio) PPhEtMA in the 5/5IL and 7/3IL mixtures, respectively a); Electrochemical voltages of the 5LCST‐5/5IL electrolyte assembled LMBs at 50, 90 and 110 °C b); Charge‐discharge profiles for a series of LCST electrolytes assembled LMBs c) and TGA curves for the 5LCST‐5/5IL electrolyte with an insertion of flammability tests in comparing with commercial EC/DEC electrolytes d).

Thermoresponsive ionic conductivity of a series of LCST electrolytes was investigated by changing polymer concentrations with a confinement of C_1_mim[TFSI]: C_2_mim[TFSI] 5:5 and 7:3 as shown in **Figure** **3**a. The highest ionic conductivity was observed in the 3LCST‐5/5IL electrolytes. Further increasing polymer concentrations from 3 to 8 wt.%, the ionic conductivity values were decreased as higher polymer contents increased the viscosities of the electrolytes and constrained the rapid mobility of ions. Similar phenomena were also observed in the issues of LCST‐7/3IL electrolytes. When the polymeric concentration was below 8 wt.%, the ionic conductivity of these LCST electrolytes were all above 10^−3^ S cm^−1^, satisfied the conduction requirements of LMBs.

To evaluate the electrochemical properties of these LCST electrolytes, linear sweep voltammetry (LSV) tests were carried out. As shown in Figure 3b, LCST‐5/5IL electrolytes demonstrated an oxidative decomposition until 5.2 V at 50 °C, while being decomposed at 5.1 V at 90 °C and 4.9 V at 110 °C, respectively. The non‐thermoresponsive 5/5IL electrolytes were also shown decreased voltages upon heating process (Figure S8, Supporting Information). It was understandable that at elevated temperatures, the electrolytes became more active to cause a narrower working voltage. Then, a battery choosing LiFePO_4_ as cathode and lithium as an anode was assembled to investigate the charge–discharge properties of these electrolytes as shown in Figure 3c. The specific capacities of LMBs were greatly dependent on various polymeric contents in LCST electrolytes. When the polymer concentrations were 8 wt.%, the specific capacities were below 120 mAh g. Much higher capacities, 134 and 139 mAh/g, were obtained by using 3 and 5 wt.% polymer electrolytes in 5/5 IL, respectively. In case of 3LCST‐7/3IL and 5LCST‐7/3IL electrolytes,the specific capacitance was slightly suppressed with much larger polarization, perhaps due to relatively lower ionic conductivities (Figure 3a). In these ternary LCST systems, polymer concentrations exhibited an obvious influence on specific capacitance. The specific capacity will be a combined result of the ionic conduction, electrolyte viscosities, and compatibility with electrodes. Thus, the 5LCST‐5/5IL electrolytes demonstrated optimized ionic conduction and specific capacity in LMBs.

The thermostability and non‐flammability of the LCST electrolytes were also important parameters to evaluate the electrolyte properties. The 5LCST‐5/5IL electrolyte is thermally stable up to 370 °C as shown in Figure 3d, and it could keep less weight loss for 20 h at 110° Cas a confirmation of non‐volatility (Figure S9, Supporting Information). Those fundamental properties suggested excellent thermostability of LCST electrolytes. Also, the LCST electrolytes are supposed to inherit the non‐flammability properties from the ILs. As shown in the insertion of figure 3d, the commercial electrolytes are easily caught fire, but the 5LCST‐5/5IL electrolyte is not flammable. This would be a dramatic safety advantage in contrast to the conventional electrolytes. Therefore, the optimal electrolyte composed of 5 wt.% PPhEtMA in 5/5 IL has desired thermoresponsive temperatures, reversible LCST behaviors, good thermostability, and non‐flammability.

**Figure 4:**
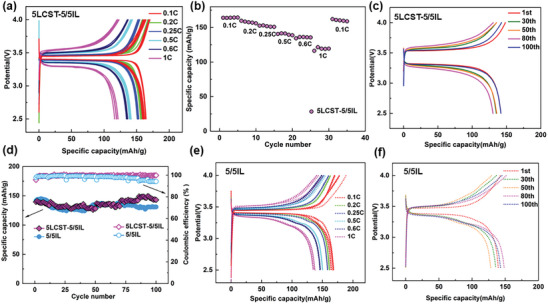
The typical charge–discharge voltage profiles of LiFePO_4_|5LCST‐5/5IL|Li smart cells with cutting‐off voltage of 2.5 4.0 V at various current rates of 0.1, 0.2, 0.25, 0.5, 0.6 and 1 C for five cycles a); Rate performance for the LiFePO_4_|5LCST‐5/5IL|Li cells b); Charge‐discharge performance at 0.5 C for 100 cycles c); Cycling performance and coulombic efficiency at 0.5 C in 100 cycles for LMBs in using 5LCST‐5/5IL and 5/5IL electrolytes, respectively d); Charge‐discharge voltage profiles for LiFePO_4_|5/5IL|Li conventional cells at various rates e) and Charge–discharge profiles at 0.5 C in 100 cycles for 5/5IL assembled LMBs f).

The corresponding charge/discharge curves at each current density were investigated with a comparison of non‐thermoresponsive 5/5IL electrolytes. The 5LCST‐5/5IL electrolyte delivered the initial discharge and charge capacities of 165 and 169 mAh g at 50 °C, respectively as shown in **Figure** **4**a. The voltage difference between charge and discharge is ≈70 mV, due to small polarization based on desired mobility of Li^+^ ions. At each current rate, the discharge capacities are 164, 156, 152, 140, 135, and 120 mAh g at 0.1, 0.2, 0.25, 0.5, 0.6, and 1C, respectively (Figure 4a). When the charge‐discharge rate returned to 0.1 C, the discharge‐specific capacity can be restored to 164 mAh g as shown in Figure 4b. The assembled LMBs demonstrated excellent electrochemical properties at various charge and discharge rates. When cycled at 0.5 C, the cell could deliver a discharge‐specific capacity of 140mAh g^−1^. Even after 100 cycles, the discharge‐specific capacity could retain 139 mAh g (Figure 4c). The specific capacity retention was as high as 99% and the average columbic efficiency was up to 99.7% (Figure 4d). The notable property of LCST electrolytes were originated from non‐thermoresponsive 5/5IL electrolytes. As shown in Figure 4e, the 5/5IL electrolyte assembled LMBs could work well at each current and discharge capacities were 166, 162, 158, 150, 146, and 137 mAh g at 0.1, 0.2, 0.25, 0.5, 0.6, and 1C, respectively. Those values were slightly higher than the corresponding LCST‐5/5IL electrolytes. It was understandable that polymer addition would increase the viscosity to suppress ion transport. After 100 cycles at 0.5 C, the specific capacity of non‐thermoresponsive LMBs was 139 mAh g, with a retention of 93% (Figure 4f), which was slightly lower than the 5LCST‐5/5IL electrolytes (Figure 4d). Also, the average coulombic efficiency of the 5/5 IL electrolyte was 94.3% (Figure 4d), which was lower than that of LCTT‐5/5 IL electrolytes (99.7%). It might be originated from the physical molecular interplay between lithium salts and PPhEtMA as confirmed by FT‐IR spectra. The present 5LCST‐5/5IL assembled LMBs demonstrated a desired charge/discharge capacitance and an improved stability of LMBs.

**Figure 5:**
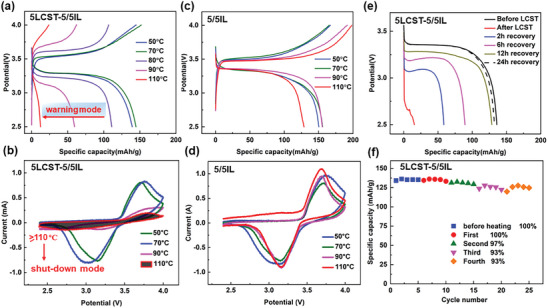
Thermoresponsive charge–discharge voltage profiles of LiFePO_4_|5LCST‐5/5IL|Li smart cells with cutting‐off voltage of 2.5 4.0 V at 50 °C, 70 °C, 80 °C, 90 °C, and 110 °C and 0.5C a); CV curves of LiFePO_4_|5LCST‐5/5IL|Li cell at 50 °C, 70 °C, 90 °C, and 110 °C b); Thermoresponsive charge‐discharge voltage profiles of LiFePO_4_|5/5IL|Li smart cells with cutting‐off voltage of 2.5 4.0 V at 50 °C, 70 °C, 90 °C, and 110 °C and 0.5C c); CV curves of LiFePO_4_|5/5IL|Li cell at 50 °C, 70 °C, 90 °C, and 110 °C d); Recovered discharge capacities for 2, 6, 12 and 24 h at 0.5C and 50 °C for LiFePO_4_|5LCST‐5/5IL|Li smart cells after overheating at 110 °C e); Reversible discharge‐specific capacity after overheating for four cycles f).

The thermoresponsive capacitance of LCST electrolytes‐based LMBs was then investigated to assess the overheating protection of smart LMBs. Battery performances of LiFePO_4_|5LCST‐5/5IL|Li at 50, 70, 80, 90, and 110 °C were explored. Thermoresponsive battery can charge–discharge normally below 70 °C, but increasing temperature gives a decline in discharge capacitance (Figure S10, Supporting Information). As shown in **Figure** **5**a, the specific capacity decreased from 161 to 59 mAh g when the temperature increased from 70 to 90 °C. It was apparent that LCST behavior could suppress the electrochemical reactions in LMBs upon heating process. Once the temperature reached 110 °C, the cells were hardly charged or discharged with a minimal specific capacity (less than 13 mAh/g). Figure 5b demonstrated temperature‐dependent CV curves of the smart cells. High temperature gave very limited charge capacitance, which was in consistent with observation in Figure 5a. The oxidative and reduction peaks at 90 and 110 °C were not obvious and the potential difference between the two peaks became large, which indicated large resistance and poor contact between electrolyte and electrodes, owing to the occurrence of LCST. In contrast, in using conventional non‐thermoresponsive 5/5IL electrolyte, the discharge capacitance continuously increased from 136 mAh/g at 50 °C to 156 mAh/g at 90 °C at 0.5 C as shown in Figure 5c. Above 110 °C, the battery became unstable and afforded a reduced discharge capacitance of 126 mAh/g. It seemed that even for neat IL electrolytes, the LMBs were not so stable at 110 °C as degradation of LiFePO_4_ occurred in cells at high temperatures.^[^ ^31^ ^]^ CV curves of the LiFePO_4_|5/5IL|Li cell were shown in Figure 5d. The currents of oxidative and reduction peaks gradually became larger, as an indication of accelerated kinetics of lithium insertion reaction at high temperatures. Compared with Figure 5b, it was apparent that LCST behavior could suppress the specific capacity of the cells at elevated temperatures. To the best of our knowledge, there is no report of thermo‐reversible electrolytes that provide mild thermal modulation of capacitance at an earlier temperature range of SEI decomposition in LMBs. It is quite different from the reported one‐step shutdown of self‐protection LMBs.^[^ ^17^ ^]^ A mild control of LMBs starting from the initial stage of thermal runaway would give more time for taking action in practical operations and greatly delay the occurrence of thermal runaway. As shown in Figure S10 (Supporting Information), the smart LMBs automatically made a mild and negative effect on specific capacity above 70 °C. In thermal runaway stage, temperatures below 100 °C are always corresponding to an initial decomposition stage of SEI membrane. It is a very early stage of thermal runaway. The present LCST cell would automatically suppress the kinetics of chemical reactions inside the cell in time while releasing less thermal energy to stop the rapid increase of cell temperatures. Such thermoresponse at 80 °C for LMBs could sense the abnormal states in advance and present a self‐adopted defense to prevent thermal runaway. Therefore, such responsive action is defined as a warning stage.

Then, high temperatures, above 100 °C, are known as critical dangerous temperatures, which is close to reach *T*_1_ of thermal runaway.^[^ ^14^, ^23^ ^]^ Further increasing temperatures would highly risk for triggering *T*_2_ and *T*_3_ stage of thermal runaway as a serious hazard. As long as the temperatures of the LMBs reaches *T*_1_, the charge or discharge reactions in LMB cells would be automatically accelerated as well as other side reactions, thereby the cells would be hardly recovered to conduct desired charge or discharge process at working temperatures. It is highly meaningful to stop charge or discharge process at such temperatures, which is protective for LMBs to minimize the thermal energy releasing at an earlier stage of thermal runaway. The 5LCST‐5/5IL assembled LMBs demonstrated a switch‐off mode to completely shut down electrochemical reactions at 110 °C, as suggested by little specific capacity values (less than 13 mAh/g). In short, the LCST electrolyte‐assembled LMBs demonstrated an intelligent modulation of electrochemical performances with two protection modes for thermal runaway: the warning mode and shut‐down mode.

Reversible modulation of electrochemical performance was investigated in smart LMBs. After overheating LMBs for 1 h at 110 °C, the cell was put at 50 °C to recover and the corresponding discharging specific capacities were utilized to evaluate its recovering process. As shown in Figure 5e, the LMB cell recovered from 12 to 57 mAh/g with a recovering rate of 40% in 2 h (Figure S11, Supporting Information). Further elongation of recovery time for 6, 12, and 24 h, the battery demonstrated a gradual increase in charging specific capacitance, 88, 125, and 135 mAh/g, respectively. Apparently, it takes 12 h for the cell to recover almost 93%. Such recovery time was quite different from thermoresponsive transmittance measurements as shown in Figure S12 (Supporting Information). The recovery rate in LMBs is certainly related to polymeric dissociation process, but also concerned about phase transitions of the polymer from separator/electrodes to electrolytes. The 5LCST‐5/5IL electrolyte showed good wettability with the LiFePO_4_ cathode and Li anode either before and after LCST behaviors (Figure S13, Supporting Information). Some LCST electrolytes might adhere on the interfaces of electrodes, which would take more time to recover. Further increasing overheating time for 5 or 24 h at 90 and 110 °C, respectively, the specific capacity during charge or discharging process was not so largely reduced in comparisin with the case of overheating for 1 h (Figure S14, Supporting Information). It seemed that it takes less than 30 min to shut down the LMBs. A real‐time responsive discharge process was also investigated as shown in Figure S15 (Supporting Information). The discharge voltages experienced a rapid decline when the operation temperatures suddenly changed from 50 to 110 °C for 5 min during discharge process. And it could reverse to normal working mode again at 50 °C. It means that LCST behavior of the thermoresponsive electrolytes could immediately and efficiently suppress the electrochemical reactions in the inner cells.

Furthermore, reversible specific capacity recoveries in four cycles of overheating protection were demonstrated in Figure 5f after the LMB cell was heated at 110 °C for 2 h, and then left at 50 °C to carry out the charge–discharge measurements. The discharging capacitance values remained almost identical in five cycles (Figure 5f). On the second time for overheating, the cell capacitance still demonstrated a recovery rate of 97%. On the following two cycles for overheating protection, the specific capacity retention remained above 93%. Such a reversible and stable battery performance stems from the excellent thermal reversibility of the LCST electrolytes in LMBs (Figure 2h). However, for non‐thermoresponsive 5/5IL electrolyte‐assembled LMBs, specific capacity could lose 28% at 50 °C after recovering 24 h from being overheated for 1 h at 110 °C (Figure S16, Supporting Information). It was sure that the conventional LiFePO_4_ cathode might experience some irreversible reactions at high temperatures.^[^ ^32^ ^]^ In contrast, the present LCST electrolytes assembled cells only lost 3% specific capacity. Such difference in reversible specific capacity loss might vividly suggest that LCST electrolytes could protect the LiFePO_4_ cell by shutting down reactions in cells at high temperatures. The above results suggest that our electrolyte could provide repetitive thermal protection for LMBs, which is featured by its excellent reversible protection, rather than one‐time protection.

**Figure 6:**
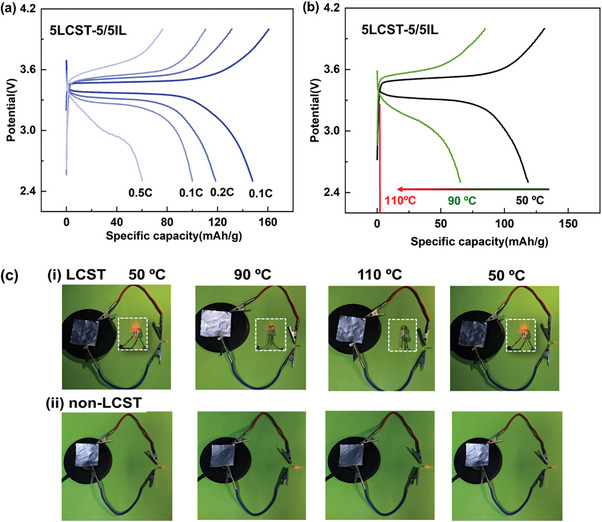
Charge‐discharge voltage profiles of LiFePO_4_|5LCST‐5/5IL|Li pouch cell at 50 °C at various current rates a); Thermoresponsive charge‐discharge voltage profiles (0.2 C) of LiFePO_4_|5LCST‐5/5IL|Li smart pouch cells with cutting‐off voltage of 2.5 4.0 V at 50 °C, 90 °C and 110 °C b); Digital pictures of LED light in using LCST pouch cells (i) and conventional pouch cells (ii) at three working modes c).

To verify the practical application of the LCST electrolytes, pouch cells were fabricated for testing thermoresponsive electrochemical performances. As shown in **Figure** **6**a, the pouch cell could be chargeable at 0.1 C with a specific capacity of 157 mAh/g. And at 90 and 110 °C in corresponding to warning and shutting‐down modes, the charge and discharge curves demonstrated thermoresponsive specific capacity change as shown in Figure 6b. The specific capacity values of the pouch cell at 0.2 C reduced from 118 to 65 mAh/g (90 °C) and 1.9 mAh/g (110 °C), respectively. The declining trend of specific capacity is in consistent with the coin cells in using LCST electrolytes (Figure 5a). It suggested that LCST electrolytes could retain three working modes either in the pouch or coin cells. Then, the lightness of a light was tested to vividly reflect LCST effects on LMBs in comparison with commercial pouch cell in using EC/DEC electrolytes. As shown in Figure 6ci, the brightness of the light was reduced upon heating process and the thermoresponsive LMBs barely output electric energy at 110 °C. However, the non‐LCST cell output the increased brightness of the LED light upon heating process as shown in Figure 6cii. The opposite brightness changes of the light are originated from the fact that LCST electrolytes could efficiently prevent the discharge process whereas the conventional one shows accelerated reaction kinetics in the cells. Afterward, the LCST pouch cell still could switch the LED on when backing to 50 °C as shown in Figure 6cii. It means that LCST electrolytes could reversibly and smartly demonstrate self‐adopted working modes according to surrounding temperatures in practical applications. Such thermoresponsive functions would be promising to improve the thermal safety of the cells as they could sense and take action in an earlier age of thermal runaway. Additionally, 5LCST‐5/5IL electrolyte was further combined with Li|LiNi0.8Co0.1Mn0.1O2 (NCM811) cathode in a pouch cell. The electrochemical performance of the assembled NCM811 pouch cell is shown in Figure S17 (Supporting Information), which also exhibits thermoresponsive specific capacity with typical three working modes. Therefore, LCST electrolytes might provide a general platform to regulate the self‐adopted cell performances to assemble smart cells.

**Figure 7:**
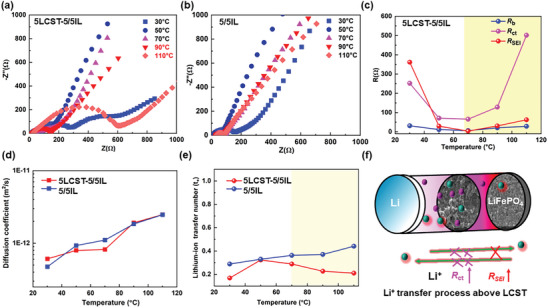
EIS curves for 5LCST‐5/5IL a) and 5/5IL b) electrolytes‐based LMBs upon heating process from 30 °C to 110 °C; LCST‐induced resistance changes upon heating process c); Thermoresponsive diffusion coefficients d) and lithium‐ion transfer number e) upon heating process for 5LCST‐5/5IL and 5/5IL electrolytes in LMBs; An illustration of thermoresponsive ion transport process f).

The mechanism of LCST suppressing the electrochemical performances in LMBs were explored by investigating ion transfer process and interfacial morphology. Electrochemical impedance spectroscopy (EIS) measurements were conducted to investigate the LiFePO_4_ battery resistances at different temperatures (**Figure** **7**a,b). The Nyquist curves demonstrated a semicircle at high frequencies and a slopping line at low frequencies, corresponding to the interfacial charge transfer resistance (*R*_CT_) on the LiFePO_4_ and Li diffusion process into cathode phase, respectively. Figure 7a clearly shows that the resistances began to reduce upon the initial heating stage. But above 70 °C, a contrasting trend was observed that the LMBs resistances abruptly increased. Non‐thermoresponsive 5/5IL assembled LMBs showed decreased resistances upon heating process (Figure 7b). It was plausible that LCST behavior was attributed to larger resistance values. The detailed resistance for charge transfer resistance (*R*_ct_), high‐frequency resistance (*R*_b_), and SEI resistance (*R*_SEI_) were analyzed as shown in Figure 7c. In LCST regions, values of *R*_SEI,_ *R*_ct_, and *R*_b_ were all increased along heating process. Those increased values were the conflicted results between LCST effect and high temperature effect on ion transfer kinetics. The noticeable increase was obtained in *R*_ct_. As shown in Figure 7c, the value of *R*_ct_ was 88 Ω at 50 °C, which predominantly increased to 520 Ω at 110 °C, due to aggregated polymers suppressing ionic charge transfer process by closing separator pores as illustrated in Figure 7f. It greatly retards the reaction kinetics across the separator and thus blocks the battery operation.

Further analysis of the impedance plots showed that the angles subtended by the low‐frequency curve with respect to x axis were higher than 60° angle below 70 °C. It meant the impedance was mainly dominated by the charge transfer process between the electrolyte and electrodes. At 110 °C, much smaller angles were observed to resemble that lithium‐ion diffusion played a major role in the impedance. The diffusion coefficient of the lithium‐ions was further calculated in using Fick's second law. Upon heating, the diffusion coefficient of lithium‐ions in the thermal‐responsive LMBs increased from 6.02 × 10^−13^ m^2^ s^−1^ at 30 °C to 2.32 × 10^−12^ m^2^ s^−1^ at 110 °C (Figure 7d). LCST showed limited capability on regulating lithium diffusion coefficient process in LMBs. It is concerned about the lithium‐ion transfer both in the separators and electrodes. It seemed that highly reactive charge transfer in the cathode at high temperatures could compensate the delay in charge transport through separators. However, Li^+^ transfer number was greatly suppressed above LCST as shown in Figure 7e. It was measured in symmetric lithium batteries with a small voltage of 10 mV. The lithium‐ion transfer number dropped from 0.32 to 0.21 upon heating process, which was not observed in non‐thermoresponsive ILs. The Li^+^ transfer number was always not so high in the ionic liquid electrolytes, owing to two cations present in the electrolytes.^[^ ^33^ ^]^ More importantly, it gave a clear proof that LCST behaviors could reduce transfer number of chargeable Li^+^, enabling a suppression of electrochemical reactions in LMBs. To conclude, LCST behavior could increase charge transfer resistance, and worsen the interface transfer process as well as reduce transfer number in LMBs as illustrated in Figure 7f. Those LCST‐induced mass transfer results agreed well with the thermoresponsive charge–discharge properties.

**Figure 8:**
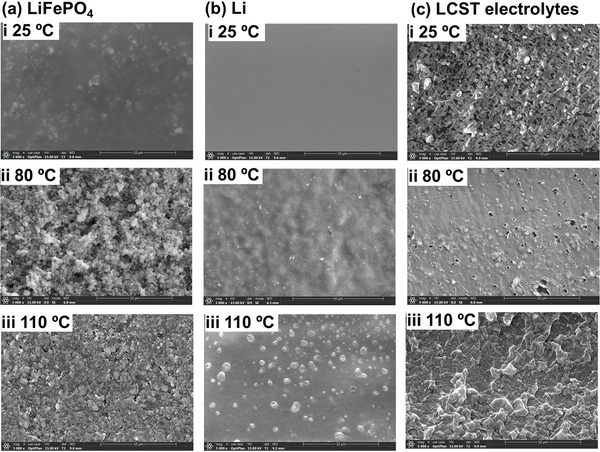
SEM pictures of the LiFePO_4_ electrodes a), Li anodes b) and LCST electrolytes c) in smart LMBs at 25 °C, 80 °C and 110 °C, respectively.

Phase separation effect on electrodes and separators were explored by scanning electronic microscopy (SEM) measurements. The electrodes and separator were not washed for a vivid observation of the LCST behavior in the cells. As shown in **Figure** **8**a, the LCST electrolytes well covered the surface of LiFePO_4_ cathode as shown in Figure 8ai. At 80 °C, polymeric particles were randomly deposited on the surface of the cathode as shown Figure 8aii. The high temperature (110 °C) caused a much heavier cover of polymer‐rich phase in the cathode. Similar deposition phenomena were also observed in the surface of lithium anode as shown in Figure 8b. At 25 °C, the lithium surface was smooth as shown in Figure 8bi. At 80 and 110 °C, aggregated polymer particles were observed on lithium anodes (Figure 8bii,iii), which reduced reactive surface for intercalation/deintercalation behavior of LMBs. Those macro‐scale deposition on the cathodes and anodes caused an apparent increase of *R*_SEI_ from 70 to 110 Ω (Figure 7c). Notably, LCST behavior of the PPhEtMA could induce open pores of the separator to be closed as shown in Figure 8ci– iii. The aggregated micro‐particles of PPhEtMA gradually fulfilled the pores of the separators, which explains large charge‐transfer resistance of LCST electrolytes and shutdown of charge/discharge process of the LMBs at 110 °C. At 110 °C, 5LCST‐5/5IL electrolytes afforded large size of particles, which were large enough to close pores of separators. Above SEM pictures vividly resembled that LCST behavior of the electrolytes could mildly control ion transfer across the separator and electrodes upon heating process. The work here provides new insights to design the thermo‐reversible and highly safety electrolytes for lithium\metal batteries in view of stimulus polymer science.

## 3 Conclusion

A non‐volatile, non‐flammable, and thermally reversible LCST electrolytes were explored with waring and shut‐down protection modes at targeted temperatures to delay or prevent the thermal runaway. The well‐designed LCST electrolytes could work normally below 70 °C with an excellent stability and desired specific capacitance. Above LCST, the specific capacitance was gradually reduced from 161 mAh/g at 70 °C to 59 mAh/g at 90 °C. The gradual suppression of electrochemical reactions was suitable to manage thermal energy in the initial stage of thermal runaway, as an advanced‐warning mode. By increasing cell temperatures to 110 °C, which is a critically dangerous temperature for thermal runaway, a strict shut down of electrochemical reactions was demonstrated to minimize the energy release. This intelligent modulation relied on particle distributions of the LCST electrolytes. Larger particles were obtained at higher temperatures, which would heavily block the pores of the separators as well as reduce reactive surfaces of electrodes, contributing to a continued deterioration of electrochemical properties. The work here provides new insights to design the thermo‐reversible and highly safety electrolytes for lithium metal batteries with warning and shut‐down mode against thermal runaway.

## 4 Experimental Section

Detailed procedures for the synthesis and characterization are provided in the Supplementary Information.

## Conflict of Interest

The authors declare no conflict of interest.

## Supporting information

Supporting Information

## Data Availability

The data that support the findings of this study are available from the corresponding author upon reasonable request.
