# Supplementary material for: Thermal Warning and Shut‐down of Lithium Metal Batteries Based on Thermoresponsive Electrolytes
Source: Adv Sci (Weinh). 2024 Jun 17;11(31):2400953. doi: 10.1002/advs.202400953 (PMC11337062; doi:10.1002/advs.202400953)
Supplement: Supplementary file 1 — Supporting Information [file ADVS-11-2400953-s001.docx]

*Supporting Information*

**Thermal Warning and Shut-down of Lithium Metal Batteries**

**Based on Thermoresponsive Electrolytes**

Yueyang Lan, Liujie Xiang, Junyu Zhou, Sheng Jiang, Yifan Ge,

Caihong Wang*, Shuai Tan, Yong Wu

School of Chemical Engineering, Sichuan University, No.24 South Section 1, Yihuan Road, Chengdu 610065 (China)

*E-mail: [wangcaihong@scu.edu.cn](mailto:wangcaihong@scu.edu.cn)

# 1.Experimental Section

## 1.1 PPhEtMA polymerization

Polymerization of phenethyl methacrylate were conducted as reported.^[1]^ Phenethyl methacrylate (5g) and AIBN (0.05g) were dissolved in 25ml 1,4-dioxane, with nitrogen bubbling for 30min. The polymerization was conducted at 70 °C for 20h. After that, PPhEtMA polymer was obtained by precipitation in using acetone and ethanol as good solvents and poor solvents, with a molecular weight of *M*_W_≈17613, *M*_W_/*M*_n_≈2.4.


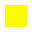

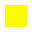


## 1.2 Electrolyte preparation

Ionic liquid electrolytes were prepared by co-solvent evaporation method, in which tetrahydrofuran (THF) was used as a co-solvent to blend 1-ethyl-3-methylimidazolium bis (trifluoromethanesulfonyl) imide salt (C_2_MIM[TFSI]), or 1,3-methylimidazolium bis (trifluoromethanesulfonyl) imide (C_1_MIM[TFSI]) with poly (phenethyl methacrylate) (PPhEtMA) and various lithium salts). In details, PPhEtMA (0.38g), LiOTf (0.936g), C_2_MIM [TFSI] (2ml) was dissolved in THF (4ml), with a strong stirring at room temperature for 24 h, and then the mixtures were dried at 70 °C for 24 h under vacuum to remove remained solvents.

## 1.3 Preparation of electrodes

LiFePO_4_ powder, super-P and PVDF solution in a mass ratio of LiFePO_4_: super-P: PVDF: 8:1:1 were mixed and milled in a ball mill at 300 rpm for 2 hours. Then the mixtures were coated on an aluminum foil collector. The LiFePO_4_ electrode was dried in a vacuum oven at 70 °C for 24 hours.

## 1.4 Battery assembly

CR2032 coin cell and home-made pouch cell were both prepared in an argon-filled glove box. LiFePO_4_ electrode, lithium sheet and porous polypropylene film (Celgard 3501, 25 μm) were used as positive electrode, negative electrode and separator to assemble coin cells, respectively. The electrolyte amount is about 40μl PPhEtMA and the assembled coin cells were sealed under a compressing pressure of 6 MPa.

# 2 Measurements and characterization

## 2.1 Characterization

FT-IR measurements were conducted by Spectrum II-10014 Fourier infrared spectrometer (Perkinelmer, The USA) with a range of 400~4000 cm^-1^ and the resolution of 4 cm^-1^. The DSC curves of LCST electrolytes were determined on a TA Q2000 differential scanning calorimeter (TA, The USA) with a temperature rate of 3°C/min upon the second heating process from -50 °C to 120 °C. Thermogravimetric analysis (TGA) was performed on a TG209 thermogravimetric analyzer (Netzsch, Germany) to obtain the thermal stability a heating speed of 5°C/min from 50 °C to 700 °C in N_2_ atmosphere. The water contact angles were characterized by OSA 60 optical surface analyzer (Ningbo NB Scientific Instruments Co., Ltd, China) at 50°C and 110°C, and each test was repeated at least twice to reduce errors. To determine the lower critical solution temperature (LCST) of the LCST-IL mixtures, transmittance was measured by USB fiber optic spectrometer NBT-4000 (Beijing Niubite Science &Technology Co., Ltd, China) with a hot stage to control temperatures ±0.3°C. The transparent solution was placed on the hot stage as covered by glasses. The transmittance curves were recorded at a heating rate of 1°C/min. Scanning electron microscopy (SEM) tests were performed on the electrode surface and separator using a field emission scanning electron microscope (Hitachi S-3400 N SEM). To determine the molecular weight of the polymer, it was tested by gel permeation chromatography (HLC-8320GPC). Electrochemical impedance spectroscopy (EIS), cyclic voltammetry (CV) test, electrochemical window test (LSV), and polarization test were measured by VersaSTAT 3-electrochemical workstation running VersaStudio software. The charge/discharge tests of the battery were carried out on the Neware CT-4008Tn-5V10mA battery test equipment. During the electrochemical characterization process, the battery temperature was controlled by a Kele temperature-controlled drying oven.

## 2.2 Electrochemical performances

The ionic conductivity of the prepared electrolytes was measured by electrochemical impedance spectroscopy (EIS) measurements in using a CHI 760E electrochemical workstation at various temperatures after rest for 1 hour. The LCST electrolytes were coated by two ITO glasses and the related measurements were performed in the frequency range of 100 mHz to 1 MHz with oscillation amplitude of 5 mV. The electrochemical stability of the LCST electrolyte was evaluated by linear sweep voltammetry (LSV) test in using an asymmetrical Li||SS battery((Li: lithium metal sheet; SS: stainless steel electrode)). The LSV test was performed using an electrochemical workstation in a voltage range of 1V to 7V at a scanning rate of 0.5 mV/s. The charge–discharge measurements were performed using a computer-controlled battery charger (CT3001A Land Battery Testing System, Wuhan, China). Before battery testing, all the coin and pouch cells were rested for 24 h at room temperature. For electrochemical performances at different temperatures, the cells were heated for 1 h at specific temperature (from 50 to 110 °C), and then the charge–discharge tests were carried out. Cyclic voltammetry (CV) tests were performed on LIBs batteries at different temperatures in the range of 2.4 V to 4.0 V at a scanning speed of 0.5 mV/ s. Temperature controlled transference number of lithium ion were measured by polarization of current.

## 3 Figures


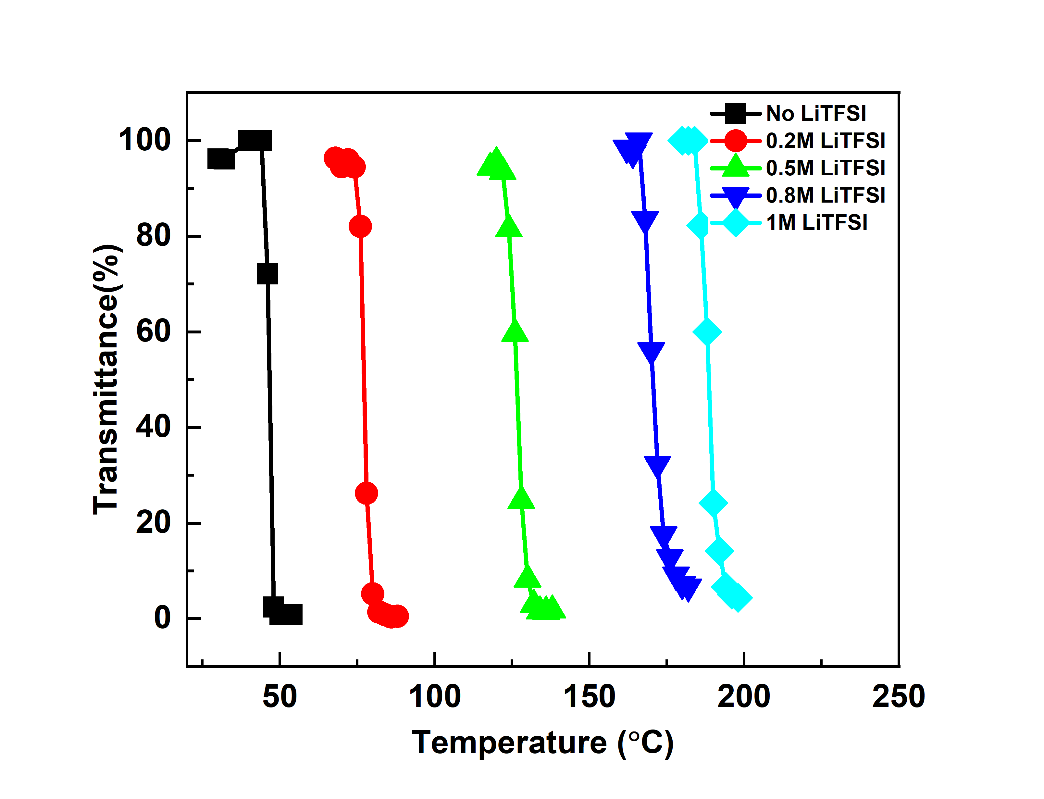


Figure S1 Transmittance measurements for PPhEtMA in C_2_mim[TFSI] containing various LITFSI salts:0, 0.2, 0.5, 0.8 and 1 mol/L.


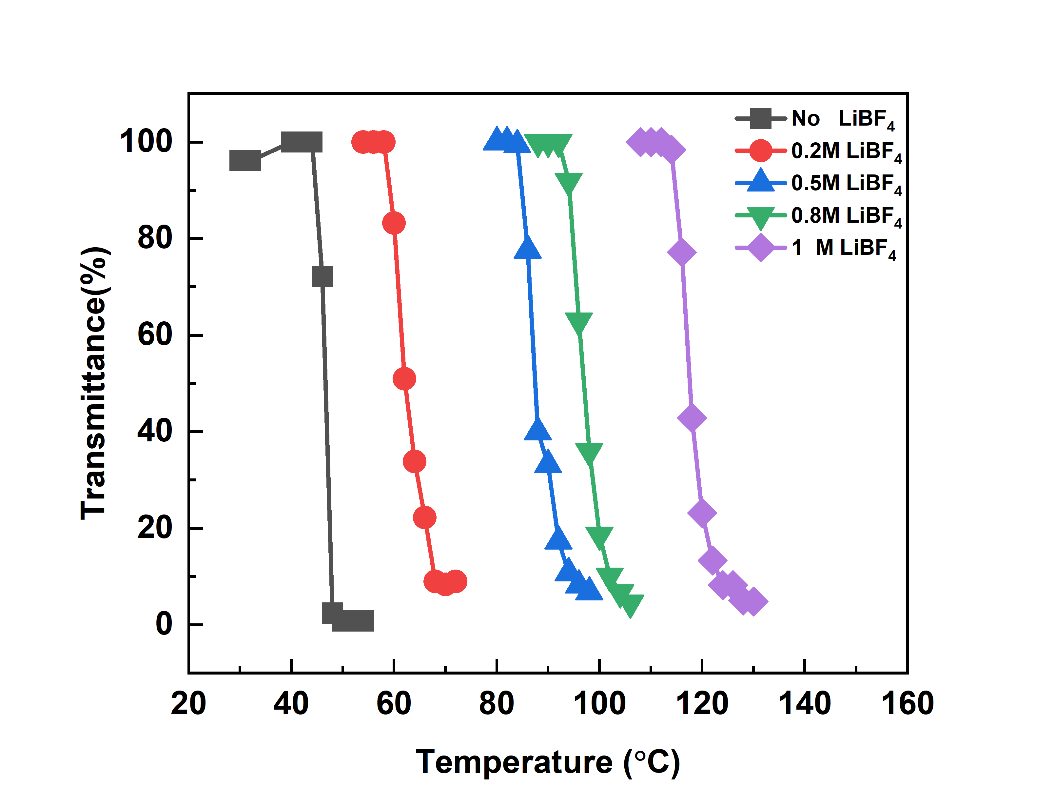


Figure S2 Transmittance measurements for PPhEtMA in C_2_mim[TFSI] containing various LIBF_4_ salts:0, 0.2, 0.5, 0.8 and 1 mol/L.


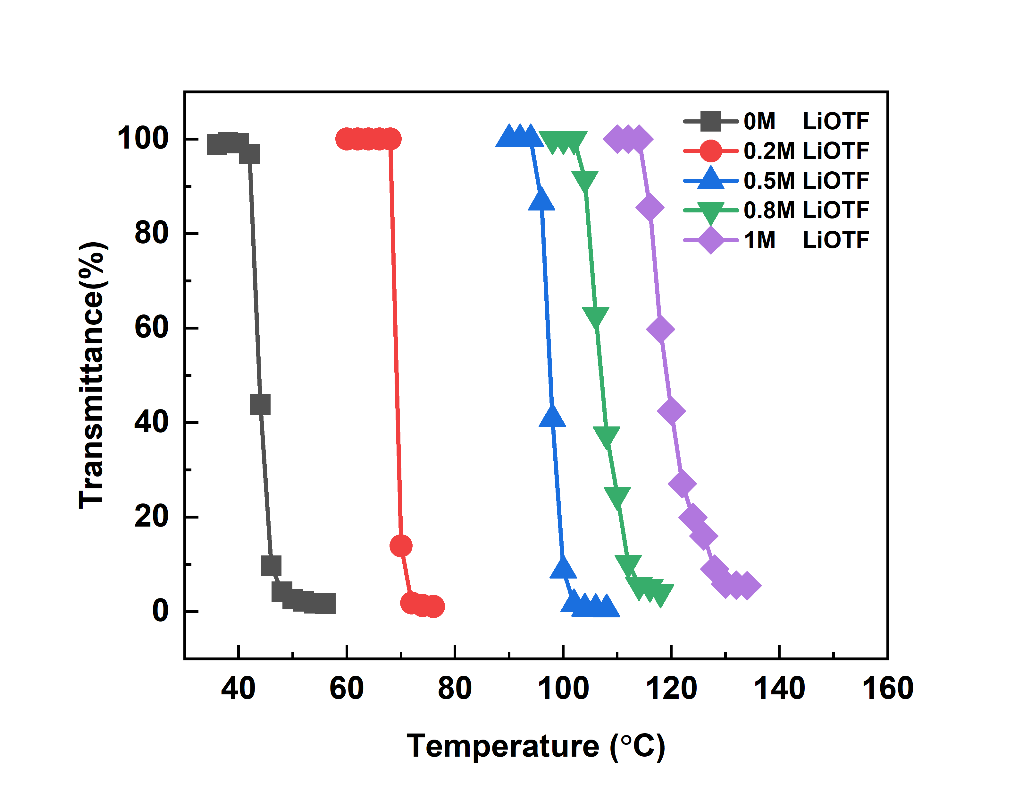


Figure S3 Transmittance measurements for PPhEtMA in C_2_mim[TFSI] containing various LiOTf salts:0, 0.2, 0.5, 0.8 and 1 mol/L.


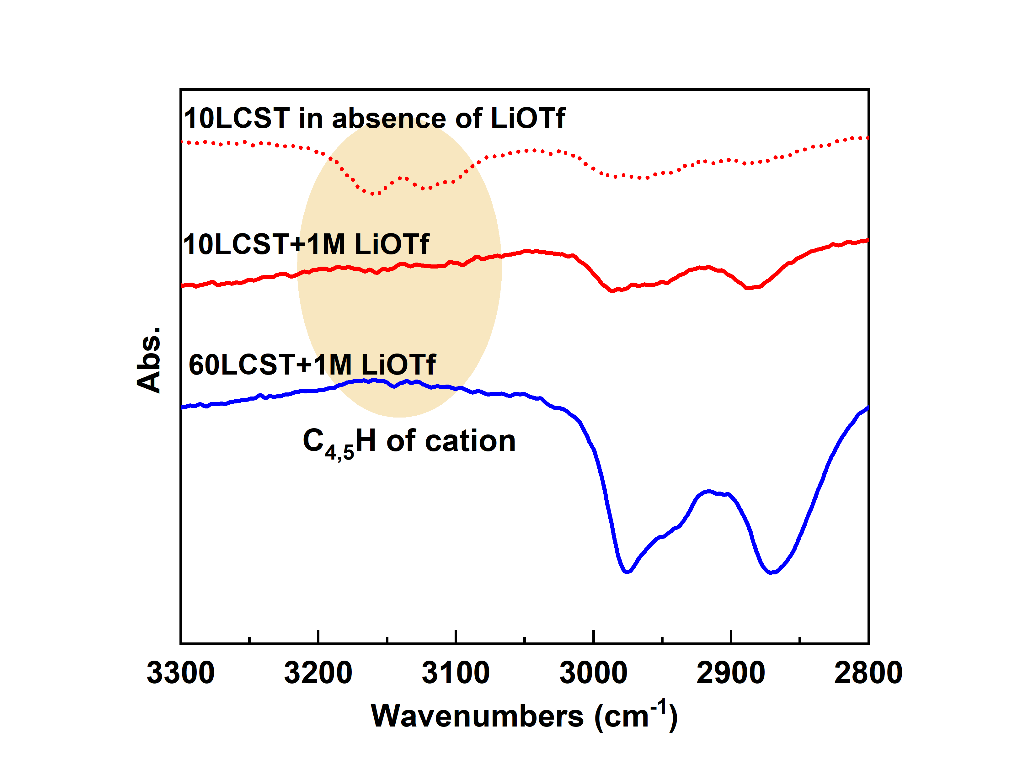


Figure S4 C_4,5_H region of imidazolium in FT-IR Spectra for LCST systems in presence and absence of LiOTf.


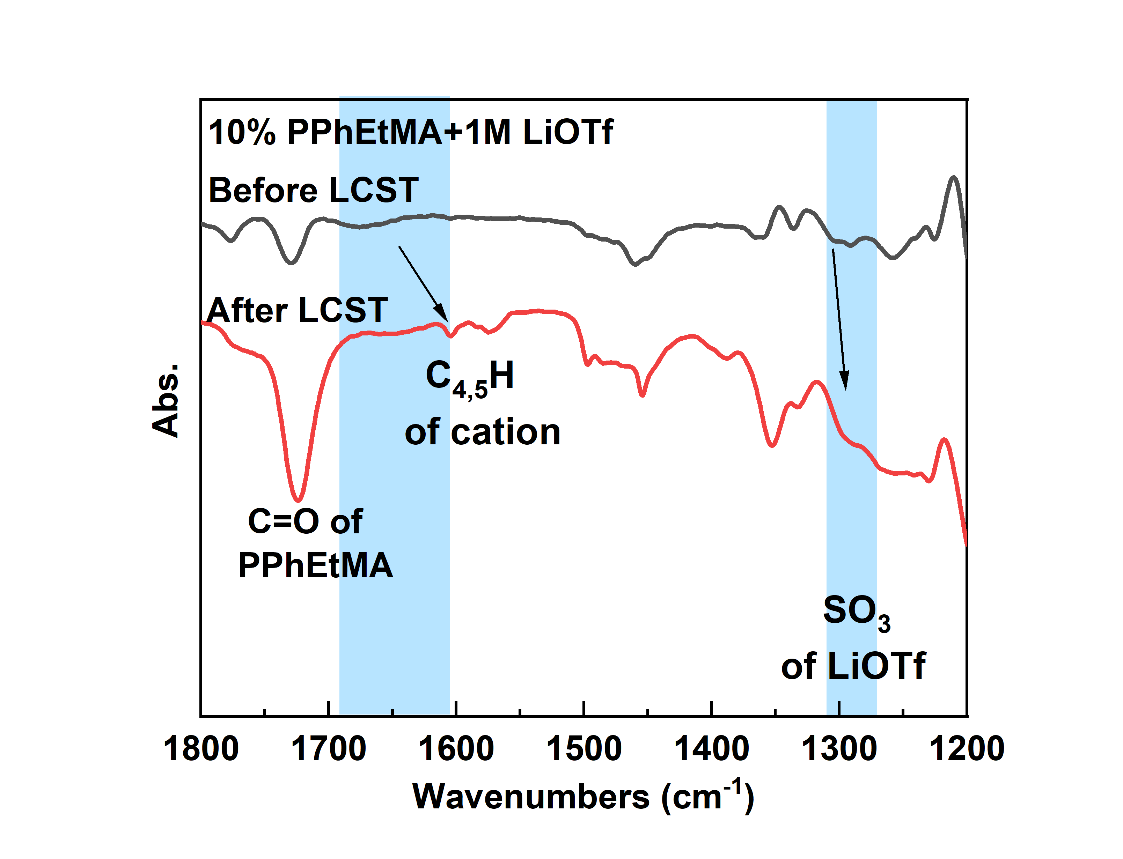


Figure S5 FT-IR spectra for LCST electrolytes before and after LCST behaviors.


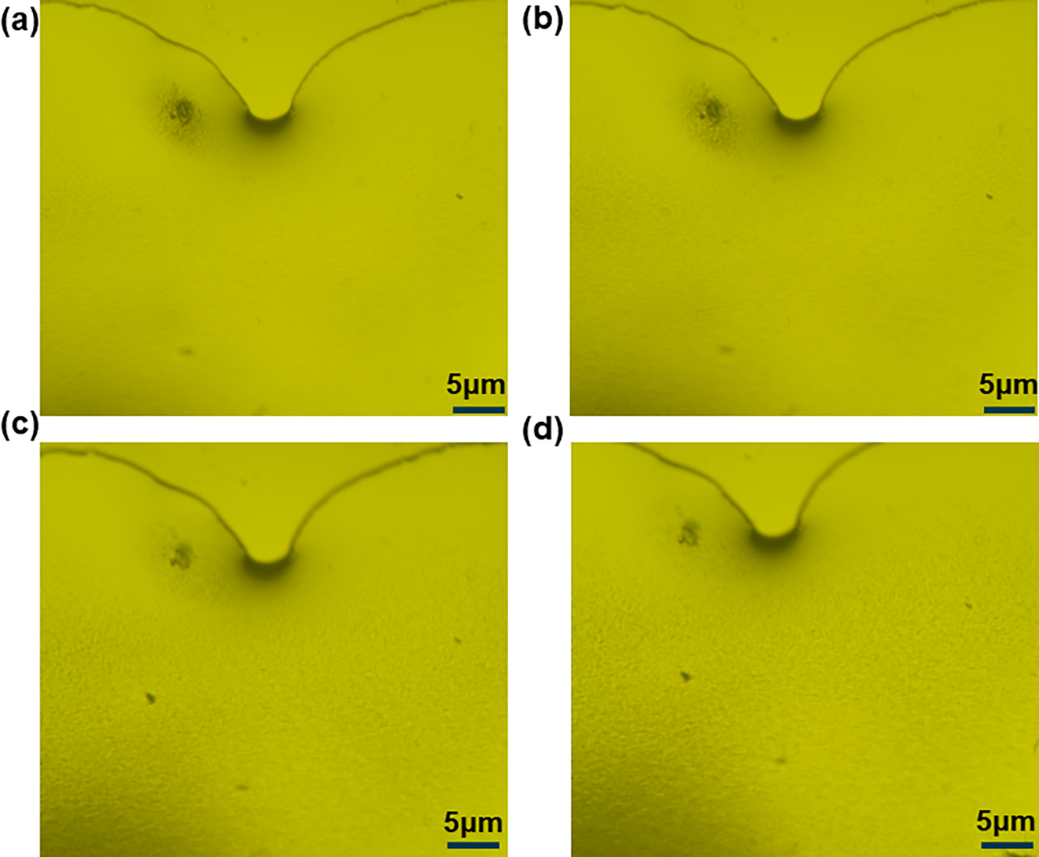


Figure S6 POM pictures of the 2LCST-5/5IL electrolyte upon heating process at 40 ºC (a), 80 ºC (b), 90 ºC (c) and 110 ºC (d).


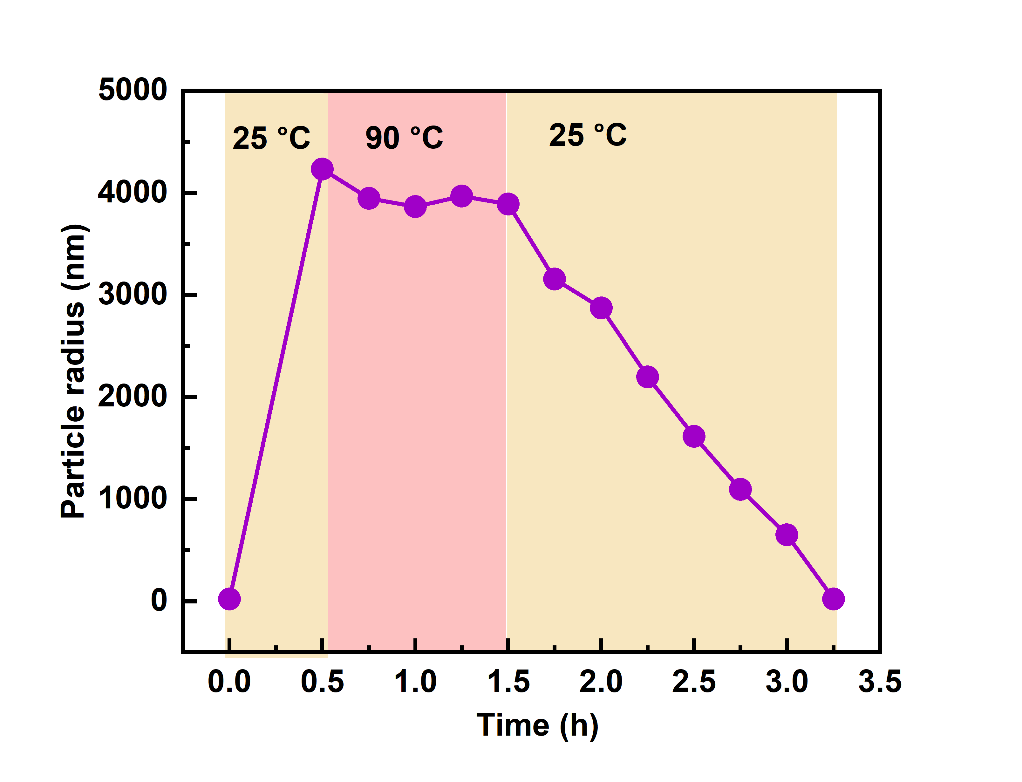


Figure S7 Size reversibility of the LCST electrolytes via switching temperatures at 25 ºC and at 90 ºC for 1 h.


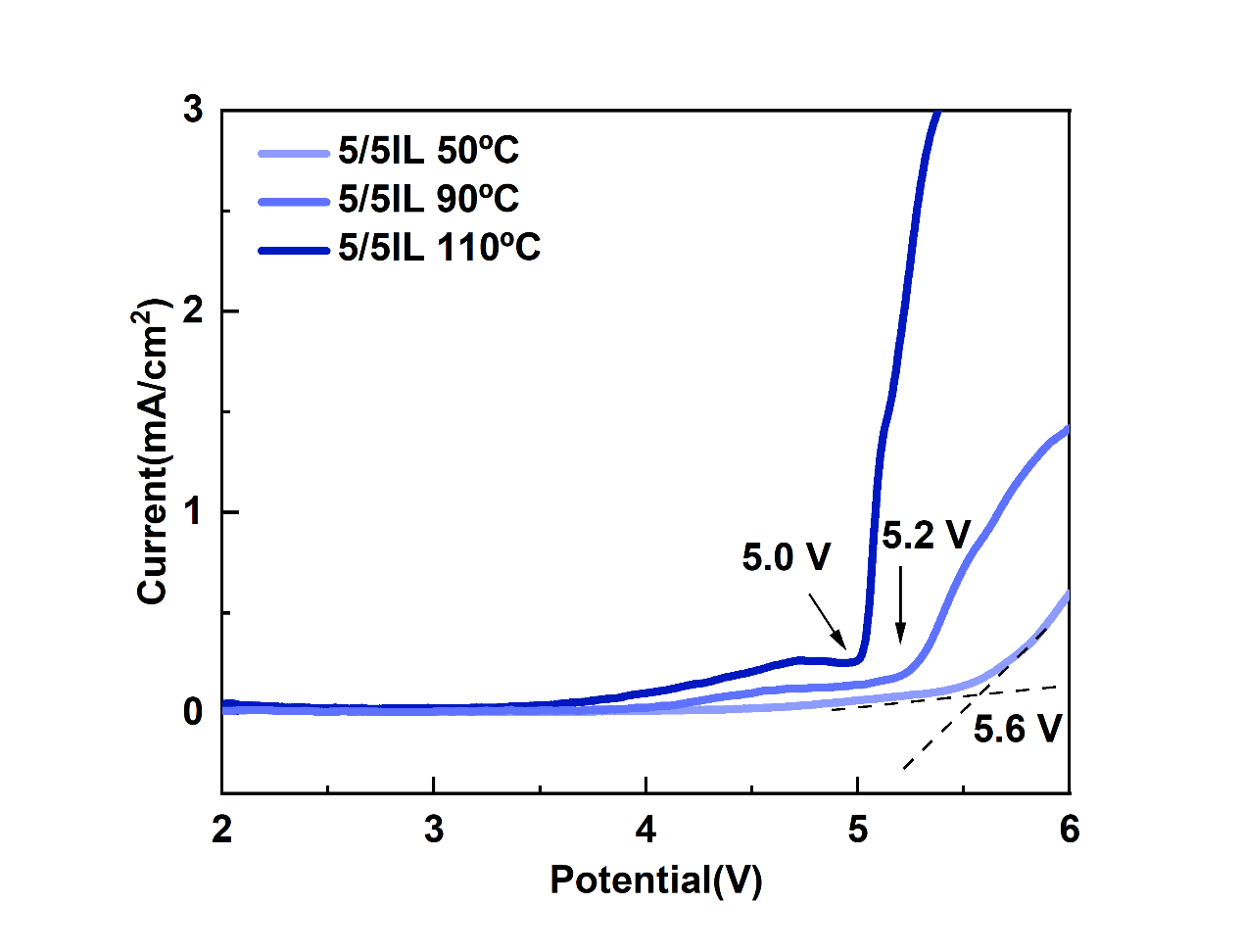


Figure S8 Electrochemical voltages of the 5/5ILs electrolyte assembled LIBs at 50, 90 and 110 ºC.


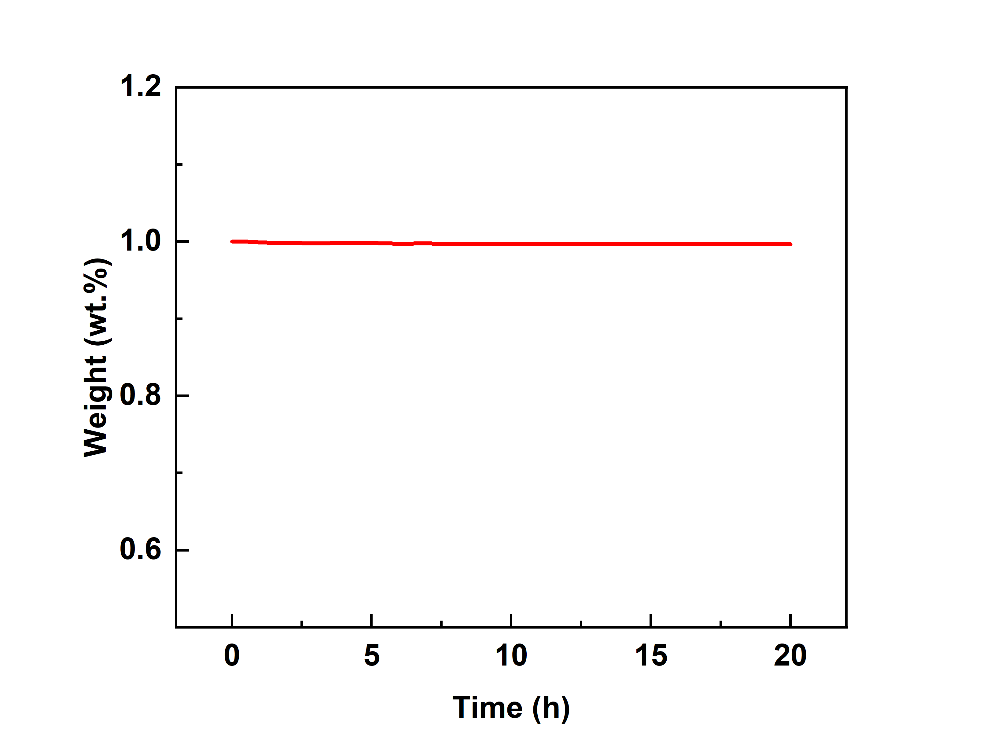


Figure S9 Weight loss for the PPhEtMA-5/5IL electrolyte at 110 ºC for 20 hours.


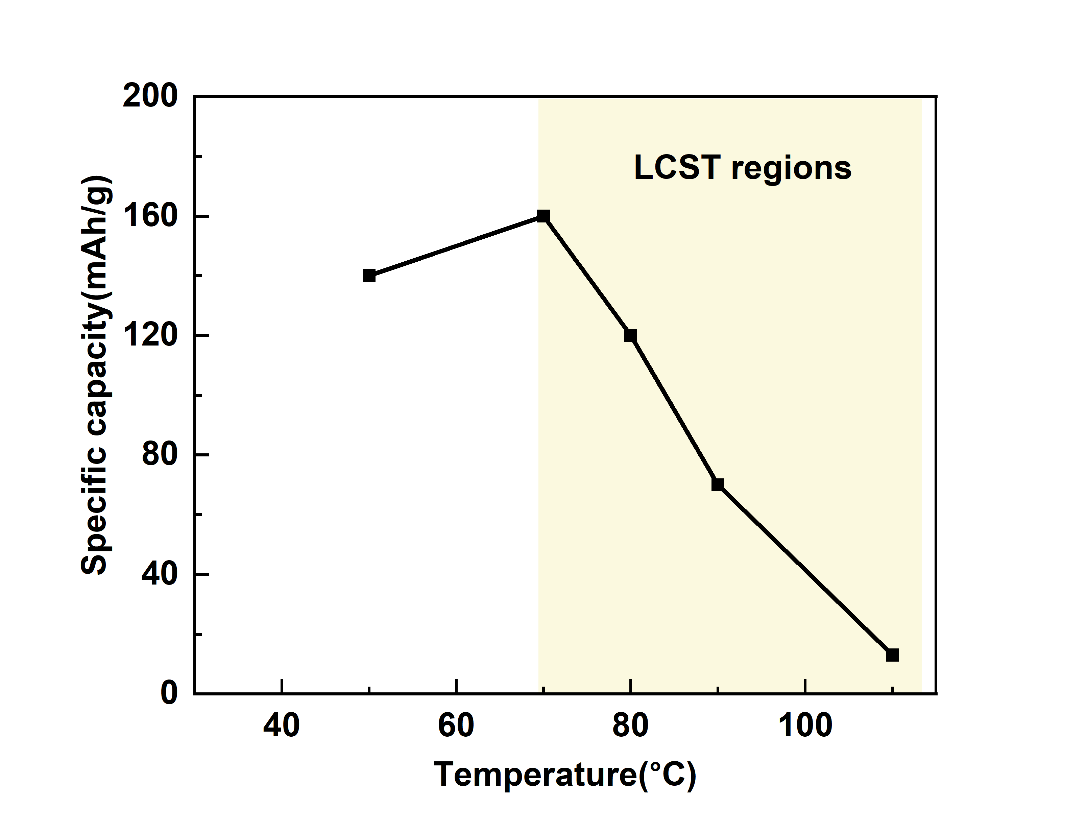


Figure S10 Temperature-dependent specific capacity of the 55LCST-5IL assembled LIBs upon heating process at 0.5C.


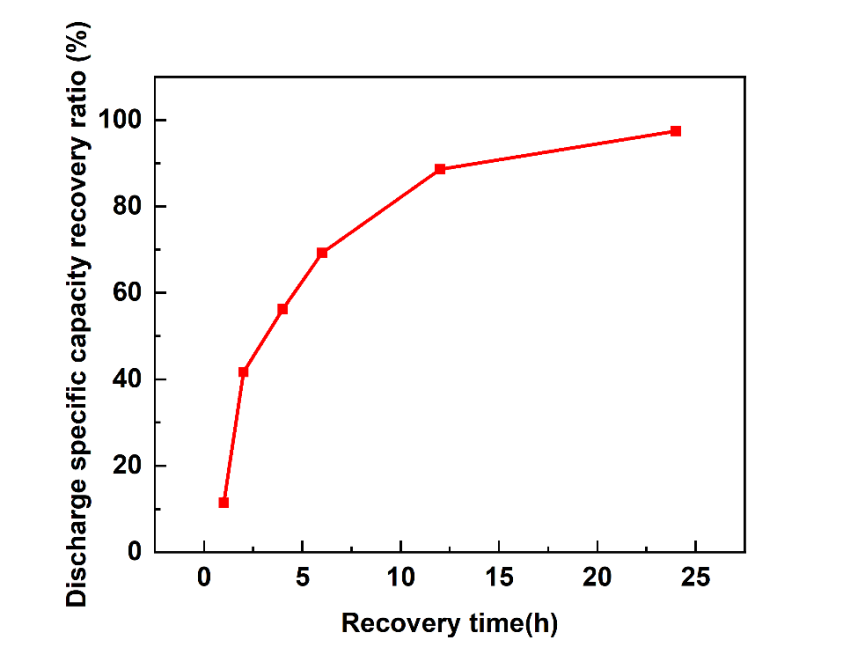


Figure S11 Recovery rate for the capacitance of 5LCS-5/5IL electrolyte assembled LIBs within 24 hours.


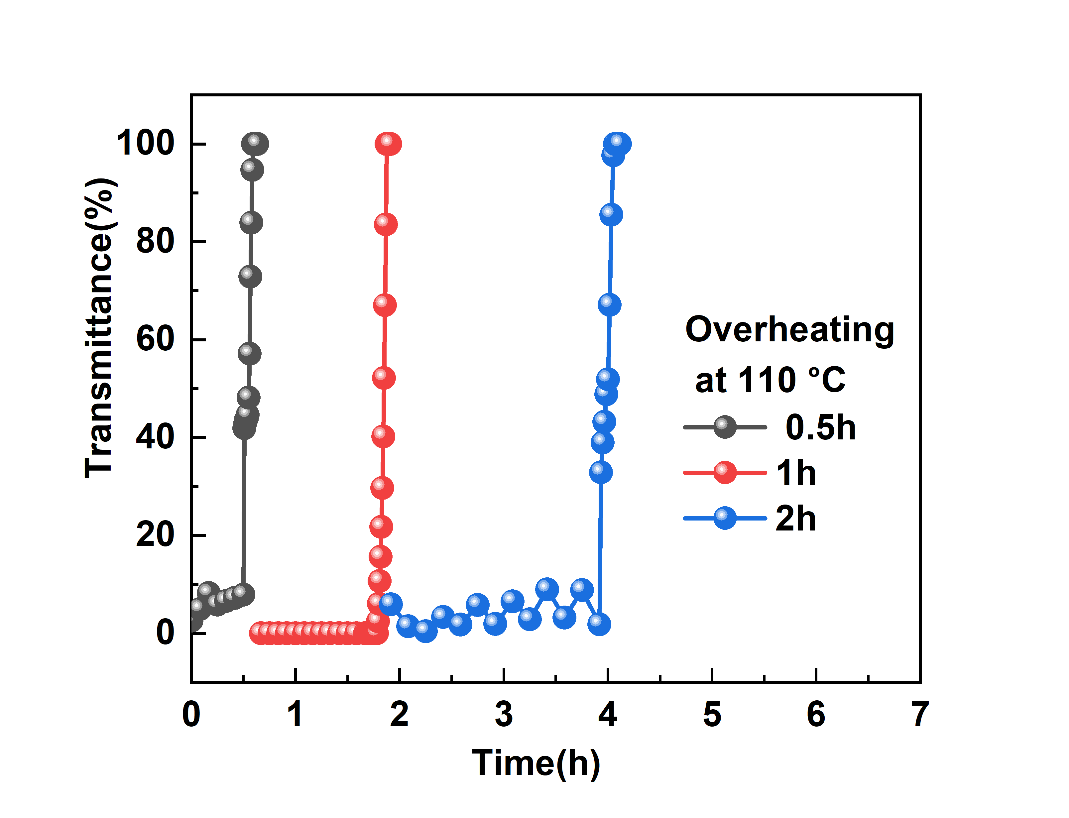


Figure S12 Reversible LCST behaviors of 5LCST-5/5IL electrolyte after being overheated at 110 ºC for 0.5h, 1h, and 2h.


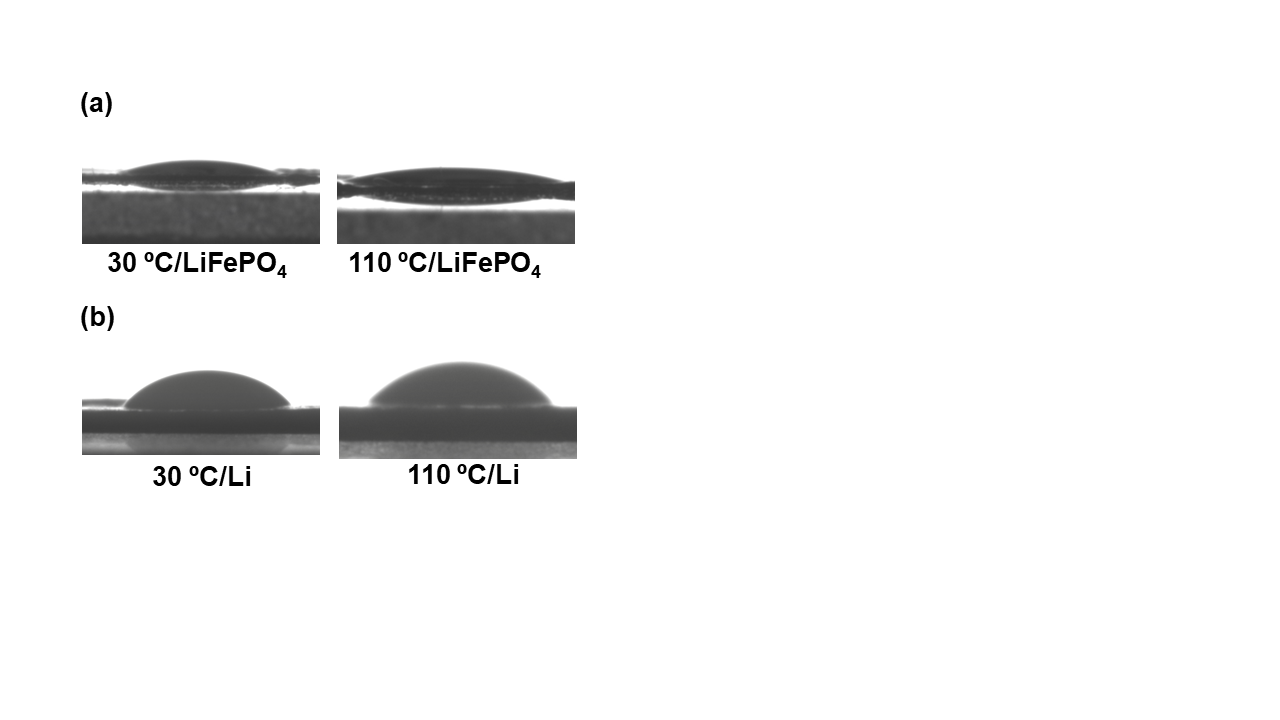


Figure S13 Wettability of LCST electrolytes on LiFePO_4_ (a) and Li (b) electrodes at 30 and 110 ºC.

**
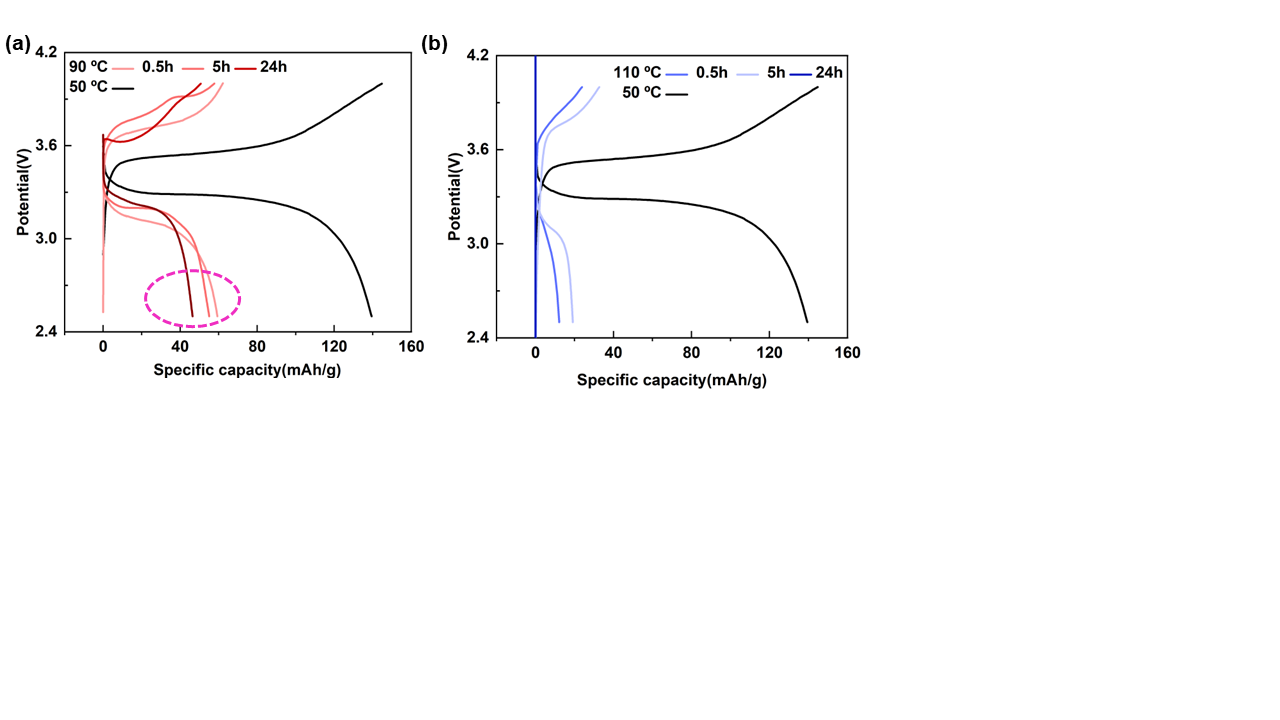
**

Figure S14 Thermoresponsive charge-discharge voltage profiles of LiFePO_4_|5LCST-5/5IL|Li smart cells with cutting-off voltage of 2.5⁓4.0V at 90 ºC, and 110 ºC and 0.5C within various overheating time.

After overheating LCST-LMBs for 0.5 h, 5 h and 24 h at 90 ºC and 110 ºC, the thermoresponsive specific capacities upon overheating process at a current rate of 0.5C were further investigated as shown in Figure 13a and Figure 13b. It is apparent that overheating time over 0.5h could result in 60% loss of specific capacities from 140 to 59 mAh/g. Further prolonging overheating time to 24 h could slightly decrease the specific values to 49 mAh/g. Similar thermoresponsive trends was also observed for the smart LMBs after being overheated at 110 ºC for different times (Figure S13b). Those behaviors suggested a highly sensitive and efficient suppression of electrochemical reactions in using LCST electrolytes.


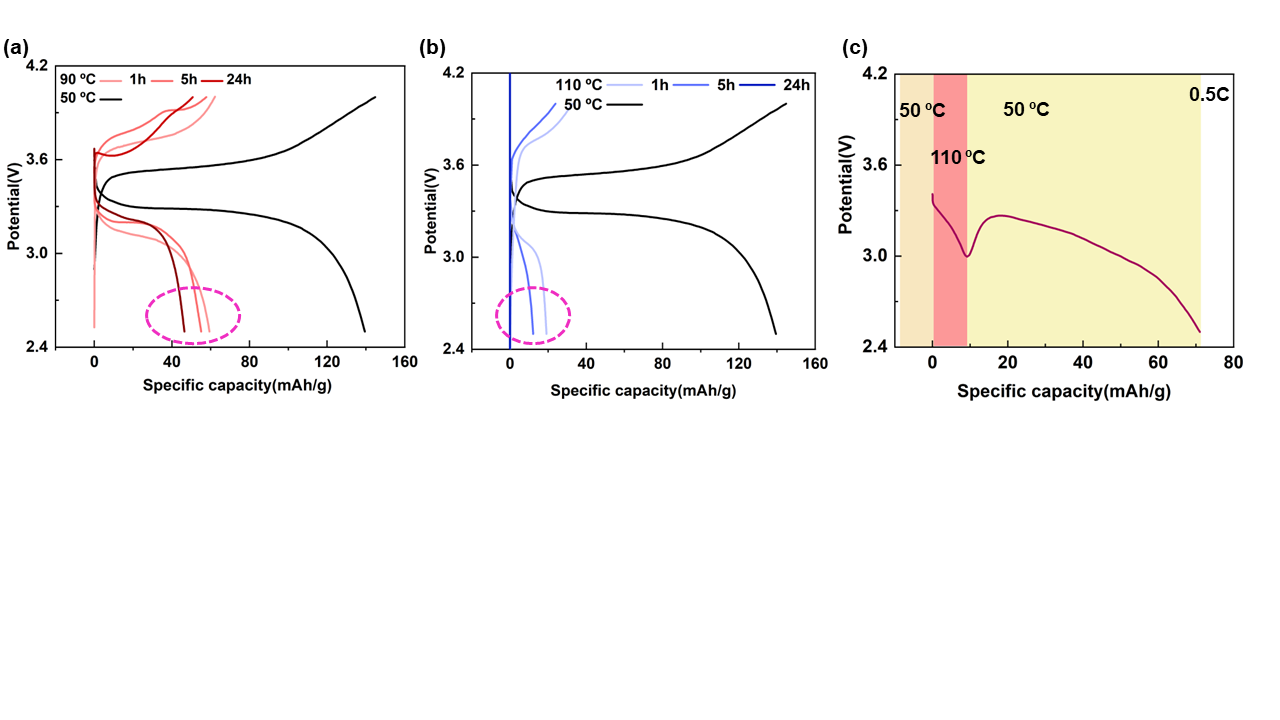


Figure S15 Thermoresponsive discharge process of LiFePO_4_|5LCST-5/5IL|Li cells with a temperature disturbance of 110 ºC for 5 min at 0.5C.

Additionally, the thermoresponsive time of LCST-LMBs was investigated by an abrupt temperature change at 110 ºC during discharging process. As shown in Figure S14, discharge voltages experienced a rapid decline when the operation temperatures suddenly changed from 50 ºC to 110 ºC for 5 min during discharge process. When the discharging temperature returned to 50 ºC again, the LMBs recovered to normal working mode. Owing to reversible LCST behaviors of 5LCST-5/5IL electrolyte, the present LCST electrolyte assembled LMB was highly sensitive towards thermoresponsive temperatures.


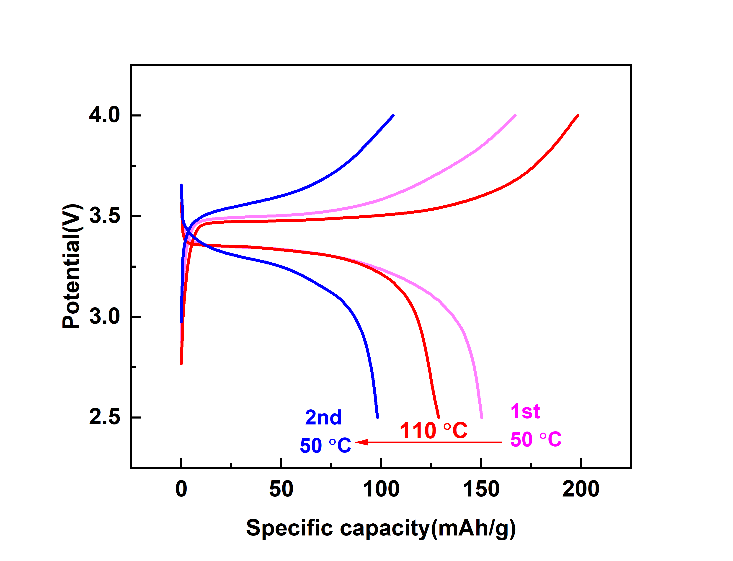


Figure S16 Reversible charge/discharge performances of 5/5 IL electrolytes assembled non-thermoresponsive LMBs by switching temperatures between 110 ºC and 50 ºC.

**
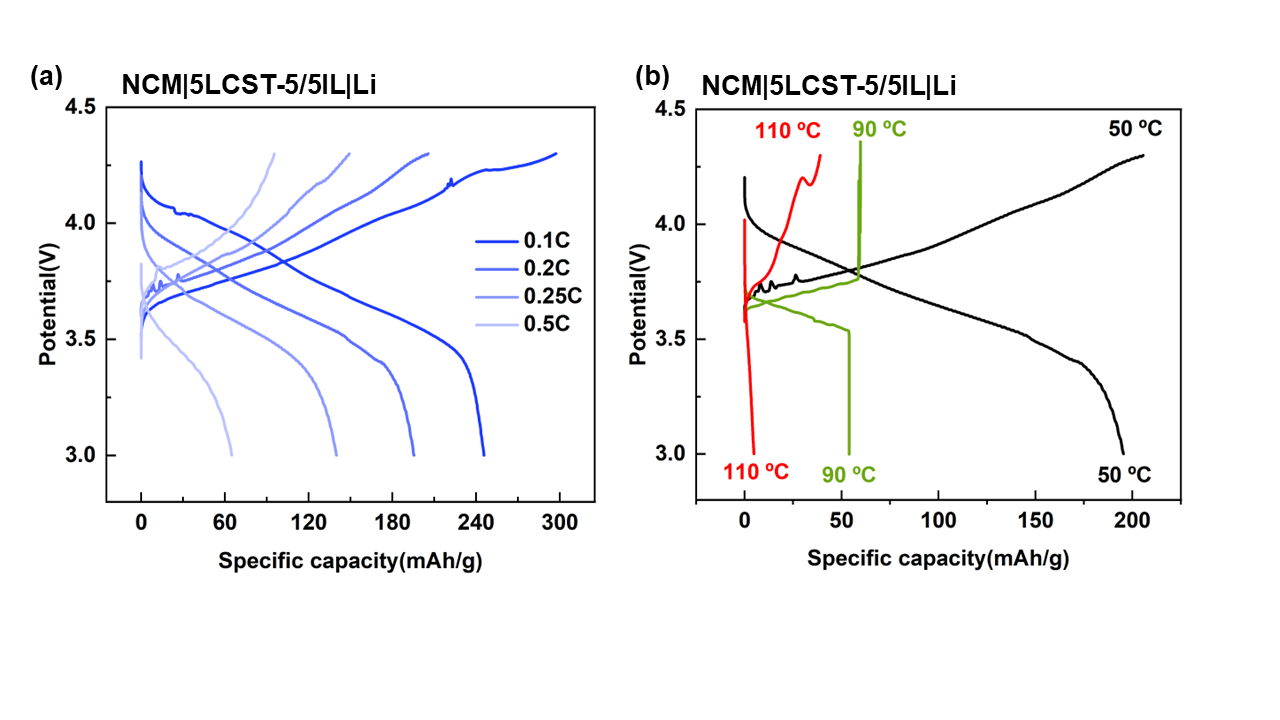
**

Figure S17 Charge-discharge voltage profiles of NCM811|5LCST-5/5IL|Li smart cells with a cutting-off voltage of 2.5⁓4.0V at 50 ºC at various current rates (a) and at 50 ºC, 90 ºC and 110 ºC and 0.2C.

**Table S1** Enthalpy values of the LCST electrolytes containing 0.2 mol/L salts as obtained from DSC curves upon the second heating process.

| Various salts  (0.2 mol/L) | LiOTf | LiBF_4_ | LiClO_4_ | LiTFSI | 0^a^ |
| --- | --- | --- | --- | --- | --- |
| Enthalpy values  (J/g of the dry polymer) | 2.26 | 2.14 | 2.47 | 2.50 | 1.48 |

^a^ means there is no lithium salt present.

**References**

[1] P. Li, S. Tan, Y. Wu, C. Wang, M. Watanabe, *ACS Macro Lett.* **2020**, *9*, 825.
